# Breaking Barriers and Bridging Gaps: Advancing Diversity, Equity, and Inclusion in Kidney Transplant Care for Black and Hispanic Patients in the United States

**DOI:** 10.3389/ti.2023.11455

**Published:** 2023-09-27

**Authors:** Chi Zhang, Amit K. Mathur

**Affiliations:** ^1^ Mayo Clinic Arizona, Phoenix, AZ, United States; ^2^ Robert D. and Patricia E. Kern Center for the Science of Health Care Delivery, Mayo Clinic, Rochester, MN, United States

**Keywords:** kidney transplant, solid organ transplant, to transplantation, diversity and inclusion, kidney allocation

## Abstract

Kidney transplantation offers better mortality and quality of life outcomes to patients with end-stage renal failure compared to dialysis. Specifically, living donor kidney transplantation is the best treatment for end-stage renal disease, since it offers the greatest survival benefit compared to deceased donor kidney transplant or dialysis. However, not all patients from all racial/ethnic backgrounds enjoy these benefits. While black and Hispanic patients bear the predominant disease burden within the United States, they represent less than half of all kidney transplants in the country. Other factors such as cultural barriers that proliferate myths about transplant, financial costs that impede altruistic donation, and even biological predispositions create a complex maze and can also perpetuate care inaccessibility. Therefore, blanket efforts to increase the overall donation pool may not extend access to vulnerable populations, who may require more targeted attention and interventions. This review uses US kidney transplantation data to substantiate accessibility differences amongst racial minorities as well as provides examples of successful institutional and national systemic level changes that have improved transplantation outcomes for all.

## Current Healthcare Delivery Challenges in Kidney Transplantation

In patients with end-stage renal disease (ESRD), kidney transplantation (KT) affords improved survival, quality of life, and overall cost advantages over other forms of renal replacement therapy such as dialysis. Patients on dialysis who remain on the waiting list have a 16.5% annual death rate, compared to 1.2% in patients who underwent KT. With further follow up, there was a 50% reduction in the 5 year mortality rate after KT compared to patients who remain on the waiting list [1]. Specifically, living donor kidney transplantation (LDKT) is the best treatment for ESRD, since it offers the greatest survival benefit compared to deceased donor kidney transplant (DDKT) or dialysis and reduces time spent on the waiting list. In addition to mortality benefits, KT also offers financial advantages. The current annual costs of dialysis are approximately $80,000 per patient per year compared to KT, which costs $30,000 per patient per year if the first-year costs are amortized over the recipient post-transplant lifetime [2, 3]. Despite clear benefits, only 3% of patients receive preemptive transplantation, including LDKT and DDKT, while the remaining initiate maintenance dialysis [2].

Though the United States has one of the most successful KT programs worldwide, as of the end of 2022, nearly 100,000 people await kidney transplantation in the US. Organ scarcity leads to a significant disparity between the demand and supply of organs as there were only 19,636 DDKT and 5,863 LDKT in 2022 [4]. While this difference is striking, the demand for organs is likely underestimated when one considers the entire continuum of care as a patient’s path towards KT requires a referral from a nephrologist, timely transplant evaluation, multidisciplinary decision regarding transplant candidacy, and time spent on the waiting list. For example, though there were approximately 560,000 patients on dialysis for ESRD in the US by the end of 2022, data suggests that failure to proceed towards transplantation is related to stagnation along any of the numerous steps in the transplant process, as only 13% of patients on dialysis were waitlisted [2, 5].

In addition to logistical barriers related to a necessarily careful evaluation process and negotiating the disease progress towards ESRD, individual barriers such as unemployment, female sex, lack of knowledge in patients and providers regarding transplantation, minority race/ethnicity, and lower socioeconomic status can also limit access to KT [6, 7]. Of course, none of these negative predictive factors exist in isolation, making the current healthcare ecosystem even more difficult to navigate for certain minority groups. According to the annual data published by the National Institute of Diabetes and Digestive and Kidney Diseases, minority patients experience higher rates of ESRD compared to white patients [2]. Yet, despite ESRD being 4.3 times more prevalent in black patients, white patients are twice as likely to undergo KT. Additionally, while Hispanic patients are twice as likely to be diagnosed with ESRD compared to white patients, they are less than half as likely to be waitlisted for KT compared to white patients from similar socioeconomic backgrounds [8]. So, KT rate variations between different racial groups could also be attributed to the likelihood of being waitlisted for KT.

The acknowledgement of the ongoing organ supply and demand narrative alone is inadequate as there are complex undercurrents that drive persistent care disparities. The process of providing more equitable care necessarily involves the understanding of disparities in current transplant care delivery using robust national and institutional data, defining disparities, and leveraging this knowledge to provide improved outcomes for all. These interventions should be aimed at providing resources to improve access, education about donation and transplantation, and to support patients before, during, and after surgery. Therefore, targeted interventions are necessary to improve equity for potential transplant candidates, their potential living donors, family members, and caregivers. Of course, changes are not one-size-fits all, so it will be necessary for individual institutions to tailor solutions to their unique patient demographics and adapt to the ever-changing healthcare landscape through the lens of quality improvement.

## The Problem: Understanding Disparities in Current Practices

Improving access to LDKT is the most reliable solution for ESRD patients. In addition to its survival benefit that exceeds DDKT and shortened waiting time, it improves access for all patients by expanding the donor pool. Increasing LDKTs could potentially address allograft access issues overall as the use of extended criteria organs have only modestly increased the donor pool and living donation would provide a higher potential source of healthy organs [9]. However, similar to other barriers to transplantation, minority patient access to LDKT is also limited compared to majority counterparts [10]. To inform effective interventions, we must first elucidate the specific barriers experienced by minority groups, as specific cultural beliefs, language barriers, and financial hardships all contribute to access issues.

### Cultural and Educational Barriers

Provision of culturally competent care for ESRD patients requires addressing beliefs that may affect transplant candidacy, recruitment of living donation, and providing education for the entire transplant process. Though outpatient dialysis centers interact with patients multiple times each week, there is large variation in referral rates between different facilities to transplant centers [11, 12]. For-profit dialysis are 50% less likely to place referrals, and nephrologists at for-profit institutions were 60% less likely to provide transplant education, citing the lack of financial incentives in time-restricted appointments as the primary reason [13, 14]. The problem with the lack of education has been so prevalent that it has penetrated popular media, with late-night comedian John Oliver, producing a segment on the issue in 2017 [15]. However, the issue is not just in the profit margins, as the comedian suggests. Compared to physicians who serve predominantly white populations, those who primarily treat black patients report spending less time on LDKT education, which is further exacerbated by the higher rates of denial regarding the need for organ transplantation in these patients [16, 17]. Even for patients who do undergo transplant candidacy assessments, black patients have protracted evaluation times due to additional testing, longer dialysis to waitlisting time, lower pre-emptive transplant rates, and a lower rate of pre-transplantation evaluation completion [18, 19]. However, medical comorbidities also do not completely explain practice variations as the 30% of patients did not receive KT education tended to be older, have non-private insurance, and receive less nephrology care prior to ESRD [20]. Not receiving education regarding KT is associated with a 53% lower rate of any access to transplantation and a 65% lower rate to LDKT, specifically. In the same study, being black was associated with a 27% increased rate of being deemed psychologically unfit for KT, a 24% lower rate of transplant care access, and a 64% reduction in the rate of LDKT access.

Healthcare disparities are complex systems that cannot be explained by racial motives alone. The Social Deprivation Index is a composite measure that incorporates data on income, education, employment, housing type, housing characteristics, transportation, and age of adults within each household [21]. Within the ESRD population, Hispanic (65%) and black patients (57%) experienced higher levels of social deprivation compared to white patients (21%). Additionally, patients with higher social deprivation indices tend to have more medical comorbidities [2]. It follows that part of the lack of education for minority populations could be a system level issue. If potential transplant candidates seek care late in the progression of chronic kidney disease, clinicians may be left scrambling to manage the organ failure, overwhelming the clinical interaction with more immediate medical concerns, rather than discussions about donor options or LDKT education [22]. Therefore, systematic and early conversations by primary care physicians, community nephrologists, and dialysis centers are necessary to promote kidney transplant access for both DDKTs and LDKTs [23].

The provision of education is necessary because without it, patients are less likely to inquire about KT on their own accord, with many either not knowing that KT is an available option and other patients not fully understanding that there is a difference between DDKT and LDKT [24]. In a survey of patients undergoing dialysis, over 10% of black men and 15% of black women reported experiencing racial discrimination during healthcare interactions [25]. The psychological stress as a result of systemic discrimination increases the fear of rejection and death from transplant surgery [26]. Similarly, in addition to general mistrust of the healthcare system, pervasive cultural myths and linguistic dissonance can further limit LDKT even when initial education is provided for Hispanic patients [27]. Family members need also be included in educational sessions because their cultural misconceptions and the belief that donors would have dramatically shorter life expectancies, be unable to have children, and contract kidney disease overtime can discourage LDKT [28]. Additionally, education does not just address information deficit because when asked specifically about their attitudes towards LDKT, they reported that lack of interest were primarily related to feelings of guilt and indebtedness to the donor [29]. This coupled with the cultural expectation that the potential donor should be the one to initiate the conversations make LDKT virtually impossible.

### Linguistic Barriers

Linguistic barriers can be another major obstacle that prevent Hispanic patients from accessing transplantation care, as over 70% of Hispanics in the United States come from Spanish speaking only households [30, 31]. This is particularly important given the secular trends in the US population as Hispanic-origin persons will constitute the largest population subgroup by the year 2050 [32]. Though most centers have access to language interpretation services, misunderstandings and mistranslations are common [33]. While families could aid in communication and often have the patients’ best interest, they lack adequate training, infringe on patient privacy in certain cases, and may distort information for the sake of protecting their loved ones [34]. Linguistic concordance is a key element of culturally competent care, and patient preferences should be considered, especially since there is incredible variation in English and Spanish fluency and linguistic preferences within Hispanic families [35, 36]. Additionally, same language patient-provider dyads are associated with greater satisfaction than the use of third-party translator.

Interestingly, over 85% of all LDKTs are performed in just 10 United States transplant programs. Additionally, all of these centers had multilingual physicians, with approximately half of them being proficient in Spanish [37]. Providing culturally concordant care is not only sensible, but also effective, with multiple centers that have created platforms to help address disparities in the Hispanic population requiring renal transplant. This presents challenges in the delivery of surgical and non-surgical care in large US hospitals due to a lack of personnel with the requisite clinical expertise and cultural or linguistic background.

### Financial Barriers

Living donor evaluation is a complex process and involves multiple appointments with transplant professionals, laboratory and imaging tests, and other healthcare interactions. These take valuable time and money from donors, as some of the costs are not reimbursed through medical insurance [38, 39]. While a donor’s gift can save millions of healthcare dollars spent on dialysis, individual donors incur costs related to travel, lodging, lost wages, child and dependent care [40]. These costs are magnified after donation surgery, especially if there are unforeseen complications [41, 42]. In addition to entrenched mistrust minority populations have about healthcare, potential donors from the same communities may experience similar healthcare access barriers. Undue financial burdens, fear of poor outcomes, and the cost associated with a prolonged and difficult evaluation after transplantation have all been identified as barriers to donation [43, 44]. This is particularly critical for vulnerable populations such as the Black and Hispanic populations, who have lower annual household incomes according to US data from the Department of Labor, as current trends suggest that living donation is an income-dependent process [45].

## Solutions That Work: Overcoming Practice Barriers

### Facilitating Conversations About Living Donation by Creating a Culturally Competent Transplant Program

Broaching potential donors is difficult because it involves admitting feelings of vulnerability, pride related to solving one’s own problems, and concerns over the impact on the health of the donor, and many other issues. Of course, fears about surgery, organ rejection, death, and future kidney disease for the donor are also prevalent for patients of all races [46, 47]. While being white and higher levels of education were predictive of willingness to initiate conversations, other factors such as age, dialysis status, and even prior transplants were surprisingly not predictive of patient ability to approach LDKT [48]. Initiating dialogue can be intimidating, especially without the guidance of a transplant team. Mistrust in the healthcare system, fears that the transplant may fail, and concerns about the health of donors post-donation not only dissuade patients from considering becoming living donors, but they can also lead potential recipients to reject these offers without thoroughly considering the repercussions of their decision [49].

Several transplant programs across the United States have developed culturally concordant transplant program models to address the needs of this population to optimize care of the recipient and potential living donors. These models have helped to improve care for vulnerable populations and have proven to be successful in achieving high rates of LDKTs, satisfaction with recipients, donors, and their families, in largely a cost-neutral approach for the transplant center [50]. To increase outreach, programs have built patient-centered and referring physician base by recruiting from high minority density dialysis units. At referral, patient preferences for culturally concordant education and language preferences are solicited and targeted education is directed with an emphasis on breaking down cultural barriers that may provide negative impressions of transplantation or of living donation. In a culturally concordant, language-sensitive approach, these initiatives have identified several barriers for patients including typical medical concerns, but also the possibility of financial burden, along with other cultural concerns such as future family planning, permanent disability, medical needs, and sexual dysfunction [51, 52]. Using a holistic initiative including the employment of bilingual and bicultural staff and engagement of local dialysis centers to facilitate outreach for Hispanic patients, programs were able to increase the proportion of Hispanic patients in the kidney waitlist by 90% and LDKT by 70% within the first 5 years of the program [53]. Follow up qualitative studies involving Hispanic kidney transplant outreach programs across multiple states showed that participants of Hispanic-focused outreach groups felt that the primary use of the Spanish language enhanced understanding regarding transplantation. While few patients and families had any knowledge regarding living donation before, over 97% of patients became more in favor of kidney transplantation in general as well as specifically in living donation at the conclusion of the information sessions [54].

While ensuring understanding about one’s own medical conditions is important, it is also necessary to engage family members because initiating conversations about the need to find a living kidney donor can be taboo in many cultures [48]. One way that has been successful in navigating this barrier is the creation of a separate advocate, a Living Donor Champion (LDC). Nearly anyone could be identified as an LDC for individual kidney recipients, including those who wished but were unable to donate. This program addresses the difficulty that some patients have with broaching the topic of living donation by empowering family members to do so on their behalf. This not only provides the family with the opportunity for active participation in their loved ones’ care, but also improves the chances of LDKT. The transplant center at John’s Hopkins was one of the first to start a formalized program. After receiving education about kidney failure and living donation, the LDC are provided vetted material and business cards to distribute to potential donors. At the end of the program, 25 potential donors were identified for the 15 patients enrolled when there was none before [55]. Other transplant centers have adopted similar programs and the added social media outreach to their training programs. Not only does this expand their network of potential donors, but attracted potential donors may also be younger and healthier [56]. Furthermore, LDC tempered some of the disparities seen in certain cultural groups as participation in such programs was associated with the 5–6 fold higher likelihood of a potential living donor referral regardless of race [57].

### Leveraging Financial Advantages

While providing the necessary language for both patients and for their families to communicate the need for kidney allografts could increase donor pool, donation interest could be thwarted by financial disincentives. Despite their altruism, there are significant financial barriers for both designated and non-designated living donors. Most living donors unintentionally incur out-of-pocket costs related to living donation, which can prohibit donation [41, 58]. Studies in Canada have additional shown that despite the maximum reimbursement being $5,500 in some provinces, the personal financial costs of organ donation often exceed the maximum reimbursement amount [41, 59, 60]. In the US, while it is illegal to provide compensation in exchange for donation, recipients are legally permitted to reimburse donors for the costs associated with living donation to make it financially neutral. Established in 2007, the National Living Donor Assistance Center (NLDAC) is a federally funded program that helps offset financial hardships incurred by altruistic donation and is available at all US transplant centers [61, 62]. Currently, 8%–10% of US living donors utilize the NLDAC means-tested program, which calculates reimbursement based on the recipient’s household income in the case of directed donation. This program helps defray out-of-pocket costs related to living donation, with over 75% of donors stating post-donation that they would not have been able to go forward with surgery without receiving financial assistance [3, 62, 63].

Other living donor expense reimbursement programs exist through paired kidney exchanges, state-based programs, or philanthropic resources. Living donor transplant programs and their social workers must be equipped with the knowledge of these resources to ensure that they can adequately counsel individual donors. Importantly, financial costs incurred after living donation can be reimbursed by a multitude of payers including funds from transplant programs themselves, state-based programs, insurance companies, and by the recipients themselves. The National Kidney Registry (NKR) is a nonprofit organization. It was started by a father who searched for multiple kidney exchange programs for his 10 years-old daughter. She eventually found a match, and the father went on to donate his kidney in exchange for a voucher, in case she would ever require a second KT [64]. The NKR aims to facilitate living kidney donor exchange, with data showing that patients who receive care at NKR hospitals are up to 3 times more likely to undergo LDKT [65].

### Optimizing Organ Utilization

For patients to gain access to transplantation, it is also critical for the transplant program to optimize practices to address the needs of waitlisted patients. Clinical protocols on living donor candidacy vary substantially between transplant programs with different institutions employing different clinical cutoffs for age, body mass index, family history of cardiovascular disease and medical conditions such as diabetes and hypertension [66, 67]. An important aspect of addressing disparities in healthcare delivery is to continuously re-evaluate clinical criteria used to offer surgical therapy by the program itself. For living donation, continuous engagement with national data and program data using a quality-assurance and performance improvement (QAPI) approach is required for regulatory compliance [68–70]. Programs must innovate in the development and execution of their clinical criteria to ensure they are casting the widest net and, in the case of living donation, facilitating the donor’s autonomy to help their intended recipient. In the context of LDKT, which is the best option to address renal failure, it is important to also understand programmatically its limitations in addressing disparities.

Not all who want to be living donors will safely be able to do so. For some patients, undergoing a DDKT is the next best option. However, waiting times vary substantially for DDKT across the United States, exceeding 10 years in many areas of the country and the rate of organ discard remains high at 30% despite the insufficiency in the number of kidney allografts available due to transplant center practice variations [71, 72]. Optimizing the use of all offered deceased donor organs is a difficult challenge but may be one of the best opportunities to address vulnerable populations. Fortunately, policies to improve coordination amongst different parts of the system such as the donor hospitals, organ procurement organizations, and transplant centers as well as improved national allocation protocols that prioritize extended criteria organs to centers that have demonstrated a history of using medically complex organs [73, 74]. This requires a clear understanding of clinical outcomes with certain types of donors, program growth, and development of resources across disciplines [75–77].

## Implementation of Future Solutions

Interventions to improve healthcare disparities begin with understanding the current conditions of the problems in a data-driven manner and defining the disparities subsequently. In the field of transplantation, the immediate issue is the incompatibility between a lengthy waiting list and insufficient of donors. LDKT rates are modifiable, and ensuring optimal access to these is critical. Yet, it has been stagnant over the course of decades, with most of the donors being white [4]. The identification of racial disparities in LDKT within the larger problem of high mortality on the waiting list has created opportunities to provide more equitable healthcare for patients. Multiple initiatives including having providers of the same linguistic and cultural backgrounds, educational opportunities, identification of advocates that initiated conversations on the patients’ behalf, and financial reimbursements have all helped reduce barriers among racial/ethnic minority communities that have been traditionally overlooked.

Addressing disparities have expanded the living donor pool, but further effort is needed. Racial and ethnic minority patients tend have difficulties finding matched donors due to higher rates of uncommon HLA types and antibody levels that may lead to organ rejection [78, 79]. Despite seemingly immutable biologic hurdles, an expanded network beyond individual centers of living donors have improved access to care for all patients in the form of paired kidney exchanges, especially when directed donors are incompatible. Paired kidney exchange has been designed and implemented throughout the United States and has helped overcome multiple types of incompatibility including ABO mismatch, HLA incompatibility, optimizing age-matching, eplet matching, and has ushered in novel concepts including temporal incompatibility, advanced donation, and voucher donation. This has been popularized in the lay media on television, and now accounts for more than 1,000 living donor transplants each year in the US [80–83]. Paired exchange improves access for minority patients with rare blood types and antibodies that are commonly found in these groups, staving off mortality and prolonged time on dialysis while waiting for an appropriate deceased donor [84]. For LDKT, paired exchange is transformational and has indirectly become an agent in the efforts to reduce disparities in access to transplant.

The United Network for Organ Sharing implemented a new kidney allocation system in 2014 to address ongoing racial disparities for deceased donor organ allocation. Given the numerous access barriers for disadvantaged minority patients, the new system not only prioritizes increased wait times, but also transitioned to using the first day of regular dialysis instead of the first day of listing. Additionally, more highly sensitized patients received priority points and the donor service area boundaries were also expanded. This translated to salient KT access changes as the previous KT access gap of 27% and 28% between black and Hispanic patients compared to white patients, respectively, narrowed to <5%. Additionally, the national KT rate for all patients also increased by 5% [85].

To address other ongoing racial tensions in healthcare, the National Kidney Foundation and the American society of Nephrology (NKF-ASN) has created a taskforce to re-evaluate the use of race in the estimation of glomerular filtration rate (eGFR). Previous models have included creatinine, age, gender, and race (black vs. non-black) based on the assumption that creatinine concentrations are directly proportional to muscle mass [86]. Clinically, this translated to black patients having higher eGFR when matched to non-black patients with identical serum creatinine measurements, age, and gender. The NKF-ASN task force recommendations to use only race-neutral equations for eGFR took place in 2022. In comparison to previous equations that included race in the estimation, the exclusion of race reduced bias and promoted earlier access to necessary transplant care [87].

## Outcomes and Maintenance

While improving access to LDKT and DDKT are commendable, the work continues. Repeatedly, minority patients demonstrate shorter graft survival, worse graft function, and higher rates of chronic allograft nephropathy [88–90]. Poorer outcomes are linked to several social determinants of health including education, health literacy, and employment [91–94]. Recent policies to expand immunosuppression drug coverage beyond 3 years has been a major legislative victory for the entire transplant population, but particularly for those recipients with concerning risk factors. Similar analyses that lead to innovative care and health policies are, therefore, necessary. Additionally, any changes made to the delivery of healthcare must function in a complex social system that can change in unpredictable ways [95]. One structured way is using Plan-Do-Study-Act (PDSA) cycles. These quickly and pragmatically test theories in a complex system in a way that is concordant with the scientific method as opposed to randomized controlled trials where variations are eliminated [96, 97]. It is through short reiterative testing that can detect if interventions can adapt to local context and respond to changing obstacles.

In addition to being more equipped to identify and to understand unique cultural practices within minority communities that may affect transplant decision making, transplant centers must also work to identify internal biases. A survey of stakeholders at a major transplant center that included transplant physicians, administrators, and clinical staff demonstrated that misconceptions regarding the increase of Hispanic patients was rooted in cultural misunderstandings. While stakeholders did not object to outreach efforts to this particular group, there was little awareness prior to the survey regarding the existence of racial disparities in transplant care access at all [98]. Additionally, misconceptions about this group also fuelled concerns about the financial impact of expanding access to Hispanic patients. This was, of course, dispelled by concrete evidence that over 40% of Hispanic patients had commercial insurance, which is 10% more compared to non-Hispanic whites [4].

## Conclusion

While KT, specifically LDKT, is the best treatment for ESRD, certain racial minority groups continue to experience access barriers. While new allocation and eGFR estimation algorithms have improved access at the healthcare system level, access barriers persist for black and Hispanic patients. The process of addressing disparities in transplantation begins with the definition of disparities, including the recognition of socioeconomic limitations, linguistic barriers, and racial inequities. With improved understanding, physicians can work to dispel cultural barriers that proliferate misinformation regarding transplantation and propagate knowledge of ways to offset financial disincentives to living donation to improve outcomes for all.

## Abbreviations

ESRD, end-stage renal disease; KT, kidney transplantation; LDKT, living donor kidney transplantation; DDKT, deceased donor kidney transplant; LDC, living donor champion; NLDAC, national living donor assistance center; NKR, national kidney registry; QAPI, quality-assurance and performance improvement.
